# The demography of range boundaries versus range cores in eastern US tree species

**DOI:** 10.1098/rspb.2008.1241

**Published:** 2009-02-25

**Authors:** Drew W. Purves

**Affiliations:** Computational Ecology and Environmental Science Group, Microsoft Research7 JJ Thomson Avenue, Cambridge CB3 0FP, UK

**Keywords:** biogeography, climate change, forest dynamics, mortality, range shifts, species migrations

## Abstract

Regional species–climate correlations are well documented, but little is known about the ecological processes responsible for generating these patterns. Using the data from over 690 000 individual trees I estimated five demographic rates—canopy growth, understorey growth, canopy lifespan, understorey lifespan and per capita reproduction—for 19 common eastern US tree species, within the core and the northern and southern boundaries, of the species range. Most species showed statistically significant boundary versus core differences in most rates at both boundary types. Differences in canopy and understorey growth were relatively small in magnitude but consistent among species, being lower at the northern (average −17%) and higher at the southern (average +12%) boundaries. Differences in lifespan were larger in magnitude but highly variable among species, except for a marked trend for reduced canopy lifespan at the northern boundary (average −49%). Differences in per capita reproduction were large and statistically significant for some species, but highly variable among species. The rate estimates were combined to calculate two performance indices: *R*_0_ (a measure of lifetime fitness in the absence of competition) was consistently lower at the northern boundary (average −86%) whereas Z^*^ (a measure of competitive ability in closed forest) showed no sign of a consistent boundary–core difference at either boundary.

## 1. Introduction

Global and regional distributions of plant biomes and species are highly correlated with climate and soils (Woodward 1987; Walter 2002; Archibold 2005). These correlations have long been of primary intellectual interest in ecology (e.g. Merriam 1894; Cowles 1899; Whittaker 1956), and there is now an urgent need to predict how they will respond to climate change (Lenoir *et al*. 2008). Decades of research have led to a detailed quantitative understanding of the climatic and physical factors controlling the distributions of many plant species (e.g. eastern US trees; Iverson & Prasad 1998), but there is little empirical information on the ecology underlying this control (see Hengeveld 1990, p. 2). Since the abundance of a given species in a given location depends on the demographic rates of growth, death, reproduction, immigration and emigration, the geographic variation in abundance that defines a species range must reflect geographic variation in those rates, currently and/or in the past (MacArthur 1984; Brown *et al*. 1996; Holt *et al*. 2004). Therefore, an obvious first step to understanding the historical and current determinants of species' ranges is to compare the demography at range boundaries to that within the core of the range. However, such comparisons have been carried out very rarely for any species (Geber 2008), and apparently never for trees, despite several studies of the structure of ranges (e.g. abundance profiles: Gaston 2003; Murphy *et al*. 2006; Sagarin *et al*. 2006) and some studies that document the population dynamics of tree species at their range boundaries without comparison to the core (see table 1.1 in Gaston 2003). Without explicit studies of geographic variation in demography, the most fundamental questions about the dynamics and determinants of the ranges of tree species will remain unanswered. Are the range boundaries set by changes in growth, mortality, reproduction or all three? How does the relative importance of these rates differ between boundaries set by temperature, water availability or disturbance? Without answers to these questions, it is difficult to see how models can be constrained sufficiently to make reliable predictions for how, and how quickly, the distributions of vegetation biomes or plant species might respond to climate change.

In part, the lack of studies on geographic variation in tree demography reflects the large amount of data that are required to estimate even one demographic rate for one location. Thus, integrated studies of the whole tree life cycle have been restricted to small regions (Pacala *et al*. 1996), and studies of climate or range–position dependency have been limited to particular demographic rates (e.g. growth: Kobe 1996, Jump *et al*. 2006; recruitment: Peñuelas *et al*. 2007). Fortunately, recent years have seen the appearance of very large, geographically extensive forest inventories that, together with appropriate statistical methods, can provide detailed information about the ecology and population dynamics of tree species over large areas (e.g. Canham *et al*. 2006; Purves *et al*. 2007, 2008). This study uses one of these datasets to document boundary–core differences in the demography of 19 of the most common eastern US tree species. The study was designed to address the following questions: (i) are there detectable differences in demography between the boundary and the core of species ranges? (ii) If so, which demographic rates vary, and by how much? (iii) How do these patterns vary among species, and between the northern versus the southern boundaries?

It is important to note that, for a given species, the relationship between the geographic variation in abundance (i.e. the location and structure of the range) and demography is non-trivial (Holt *et al*. 2004; and see §4). Therefore, the patterns documented here do not, by themselves, explain the geographic distribution of US tree species, nor do they contain sufficient information to develop models to predict how those distributions might change in the future (see §4). Rather, the intention was to provide one of the key sets of empirical observations that are needed to develop and verify such models.

## 2. Material and methods

I made use of a large forest inventory database (the US Forest Service Forest Inventory Analysis, FIA: Smith 2002; McRoberts *et al*. 2005) to examine geographic variation in the demography of 19 of the most common eastern US tree species. The FIA consists of a network of sample plots distributed across the forested portion of the United States. The plots are resurveyed every 5–15 years, at which time all individual trees above a threshold size within each plot are identified to species, recorded as alive or dead, and measured for stem diameter (at breast height). When combined with simple Bayesian parameter estimation methods (see the electronic supplementary material), these data allow, for any species *j* and any defined geographic region *R*, an estimation of five key demographic rates: the diameter growth rate (cm yr^−1^) of canopy trees *G*_*L*,*j*,*R*_, and understorey trees *G*_*D*,*j*,*R*_; the average lifespan (years) of canopy trees *ρ*_*L*,*j*,*R*_ and understorey trees *ρ*_*L*,*j*,*R*_; and a per capita reproductive rate $F_{j,R}^{\text{capita}}$, defined as the annual number of new recruits per unit of standing basal area of species *j* (m^2^ ha^−1^ yr^−1^). These rates can be estimated from the data because the FIA follows the fates of a very large number of individual trees. Some trees survive from the time of the first survey to the time of the second, allowing average lifespan to be estimated, and those trees that do survive increase in diameter, allowing the average diameter growth rate to be estimated (see the electronic supplementary material). The appearance of new recruits (i.e. individual trees above the threshold size appearing in the data for the first time) is also recorded in the FIA. Subject to some simplifying assumptions, the rate of arrival of these new recruits can be used to estimate per capita recruitment (see the electronic supplementary material).

Species were selected by (i) extracting all species with 10 000 trees or above recorded in the FIA data and (ii) discarding any species appearing primarily as shrubs (rather than trees), with the range centre outside the US, or with demographic rates known to have been substantially affected by disease or management (see the electronic supplementary material). The resulting list of 19 species includes conifers and broadleafs with contrasting ecology and ranges (Burns & Honkala 1990*a*,*b*), which together account for approximately 50 per cent of the individual trees in the eastern FIA data.

The analysis sought demographic differences between the core and the boundaries of species ranges. Therefore, the first step was to define, for each species *j*, three regions *R*: the core; the northern boundary; and the southern boundary. The division into these regions for species *j* was set entirely by the inventory data for *j*, as follows: (i) discard all 0.50×0.50° grid cells containing no record of *j*; (ii) rank the remaining grid cells according to the average abundance of *j* within the cell, from highest to lowest, and thereby classify the cells into three *abundance bands* (i.e. classify the first third of the cells in the ranked list into abundance band 0, the next third into abundance band 1 and the final third into abundance band 2); (iii) define the *core* of the range as consisting of all grid cells within abundance band 0, regardless of geographic location; (iv) rank the grid cells occupied by *j* according to latitude, and thereby divide the cells into three *latitude bands* (latitude band 0, northernmost; 1, middle; 2, southernmost); (v) define the *northern boundary* as consisting of all grid cells that lay both within abundance band 2 and latitude band 0, and define the *southern boundary* as consisting of all grid cells that lay both within abundance band 2 and latitude band 2.

**Figure 1:**
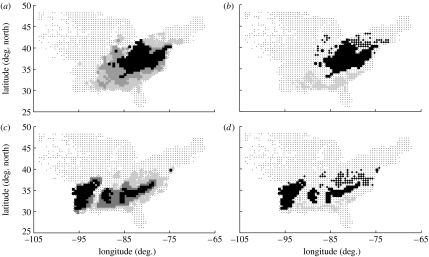
(*a*,*c*) The range of each species was divided into three abundance bands according to the average abundance within 0.5×0.5° grid cells (see §2 and the electronic supplementary material). Cells not containing the species were omitted. Abundance band 0 (the third of cells with the greatest abundance; black squares) constitutes the *core* of the range; band 2 (the third of cell with the lowest abundance; light grey squares) constitutes the *boundary* of the range (abundance band 1, dark grey squares). (*b,d*) The *northern boundary* (diamonds) of the range consists of all cells within abundance band 2, and within the northernmost third of the cells occupied by the species; similarly, the *southern boundary* (circles) consists of all cells within abundance band 2, and within the northernmost third of the cells (squares, core). The crosses show all grid cells containing at least one forest inventory plot. Two example species are shown: (*a,b*) tulip tree (*Liriodendron tulipifera*) has a unimodal core (i.e. the core is contiguous), and a relatively symmetric range; (*c,d*) shortleaf pine (*Pinus echinata*) has a multimodal core and an asymmetric range.

It is important to note that this approach to defining the core and boundaries of species ranges avoided the need to make any *a priori* assumptions about the shape or structure of species' ranges, allowing instead for the irregular, asymmetric and often multi-core ranges observed in eastern US trees (Murphy *et al*. 2006; figure 1). The use of discrete regions is for simplicity only and is not intended to suggest that geographic variation in demography is discrete. The focus on the northern versus the southern range boundaries was motivated partly by previous discussion of the (contrasting) causes of these two kinds of boundaries (e.g. MacArthur 1984; Loehle 2003); partly by the fact that, in the US (Iverson & Prasad 1998) and elsewhere (Thuiller *et al*. 2005), tree species distributions are highly correlated with measures of temperature (which in the eastern US are highly correlated with latitude); and partly by the fact that bioclimate envelope models predict potential northern movements of tree species (e.g. Thuiller *et al*. 2005; Ohlemuller *et al*. 2006), making the understanding of the causes of the northern versus the southern range boundaries of primary concern.

The second step was to estimate the five demographic rates for each of the three regions (core, northern boundary and southern boundary). Parameter estimation was carried out separately for each species and region, returning, for each rate, a most likely value and confidence intervals (see the electronic supplementary material). As a measure of the difference between the boundary and the core, I then defined, for each of the five rates, for each type of boundary (northern and southern) and for each species, a boundary–core ratio *Ω*, defined as the logarithm of the ratio of the rate in the boundary to that in the core. For example, *Ω*(*G*_*L*,*j*_, north) measures the difference between the growth rate of canopy trees of species *j* at the northern boundary of *j*, and that at the core of *j*. Error propagation was used to estimate a most likely value and 95% CI on each value of *Ω*, taking into account the uncertainty in the pair of rates used to calculate *Ω* (see the electronic supplementary material). This means, for example, that *Ω*(*G*_*L*,*j*_, north) greater than zero implies greater canopy growth rate at the boundary, *Ω*(*G*_*L*,*j*_, north) less than zero implies lower canopy growth rate at the boundary, and a 95% CI on *Ω*(*G*_*L*,*j*_, north) that does not include zero implies a statistically significant difference in canopy growth rate between the boundary and the core at *p*<0.05. A list of estimates for the value of each demographic rate for each species, within the core, the northern boundary and the southern boundary, *Ω* values for each rate at each boundary type, and confidence intervals for each of the above, is given in the electronic supplementary material.

The third step was to combine the estimates for the individual demographic rates into indices of overall performance and to examine how these indices differed between the boundaries and the cores. The first index used here, *R*_0_, is defined as the average number of new recruits produced within the entire lifetime of an open-grown tree (i.e. a tree that has never experienced shading by other trees): $R_{0,j,R}=(1/10 000)\cdot (\pi /2)\cdot F_{j,R}^{\text{capita}}\cdot G_{L,j,R}^{2}\cdot \rho_{L,j,R}^{3}$, where the factor 1/10 000 corrects for the units of $F_{j,R}^{\text{capita}}$ versus those of *G*_*L*,*j*,*R*_. The index *R*_0_ is a measure of the ability of species *j* to spread into an empty landscape. Note that *R*_0_ does not depend on understorey growth or lifespan.

However, *R*_0_ does not represent performance in the closed forests within which the majority of eastern US trees spend most of their lives. Because the height-structured competition that determines relative performance in forests is insufficiently understood, there is currently no universally accepted scheme for combining different demographic rates into a single index of performance. In this case, as a measure of performance in closed forest, I used Z^*^, defined as the canopy closure height of an equilibrium monoculture of *j*. This index is derived from the PPA model of forest dynamics (see Adams *et al*. 2007; Purves *et al*. 2008; Strigul *et al*. 2008). This work suggests that, in many circumstances, species in closed forests form a competitive hierarchy according to Z^*^. For example, Adams *et al*. (2007) showed that, in the PPA model, a monoculture of the species with the greatest Z^*^ cannot be invaded by any other species, provided species differences in canopy transmissivity are small. Purves *et al*. (2008) have shown that, in the eastern US Lake States, the observed late-successional dominant species were those with the greatest Z^*^. With a few minor additional assumptions (see the electronic supplementary material), the index Z^*^ could be calculated from the rates estimated here: $Z_{j,R}^{*}\approx \alpha {\left[G_{D,j,R}.\rho_{D,j,R}\right]}^{\beta }{\left[\text{ln}(2\pi \lambda F_{j,R}^{\text{capita}}G_{L,j,r}^{2}\rho_{L,j,R}^{3})\right]}^{\beta }$, where *α* and *β* are parameters defining height allometry, and *λ* (which equals 0.000025) is a correction factor that converts the units of $F_{j,R}^{\text{capita}}$ (see the electronic supplementary material).

The values of *R*_0_ and Z^*^ were calculated for the core, and the northern and southern boundaries, of all species. Error propagation was used to provide confidence intervals on the indices that reflected uncertainty in each of the parameters used to calculate them (see the electronic supplementary material). For *R*_0_, *Ω* values (defined as above, and complete with 95% CIs) were used to compare the values of *R*_0_ in the boundaries with those in the core. For Z^*^, *Ω* values could not be used because Z^*^ can take zero values. Therefore, for Z^*^, the measure used was the difference, i.e. the value of Z^*^ in the boundary minus that in the core. Error propagation was used to provide 95% CIs on these differences. All calculations for *R*_0_ and Z^*^ should be viewed with some caution for two reasons: (i) both indices involve $F_{j,R}^{\text{capita}}$, which was hard to estimate in comparison with the other rates (see the electronic supplementary material); and (ii) both indices implicitly extrapolate short-term average rates to large old trees, which in reality are likely to show reduced growth and enhanced mortality in comparison with an average tree.

## 3. Results

### (a) Demographic rates

**Figure 2:**
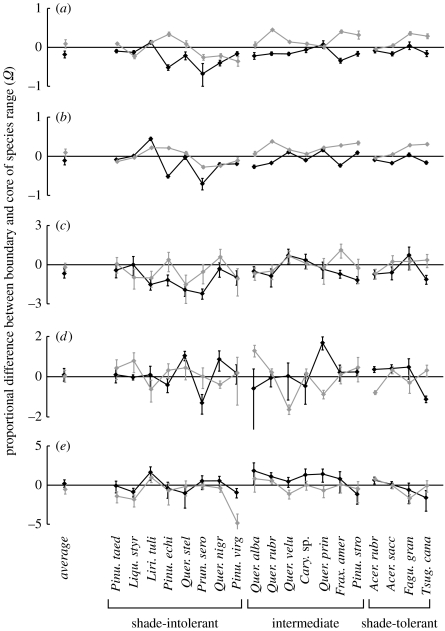
Boundary–core differences in each of five demographic rates for each of 19 common US tree species. Calculations were carried out separately for the northern boundary versus the core (black diamonds), and the southern boundary versus the core (grey diamonds). *Ω* is the log of the ratio of the rate in the boundary to that in the core. Therefore, negative *Ω* implies that the rate is lower in the boundary, and positive *Ω* implies that the rate is greater in the boundary, when compared with the core. Error bars are 95% CIs on *Ω*. Species are plotted and grouped according to shade tolerance classifications (taken from Burns & Honkala 1990*a*,*b*). Note that *Acer rubrum* is classified as ‘tolerant’, whereas the remaining species marked as tolerant in this figure are classified as ‘very tolerant’. Within shade tolerance categories, species are plotted in descending order of total abundance. *Cary* sp. refers to all hickories (genus *Carya*) combined. Otherwise species abbreviations refer as follows: *Pinu. taed*., *Pinus taeda*; *Liqu. styr*., *Liquidambar styraciflua*; *Liri. tuli*., *Liriodendron tulipifera*; *Pinu. echi*., *Pinus echinata*; *Quer. stel*., *Quercus stellata*; *Prun. sero*., *Prunus serotina*; *Quer. nigr*., *Quercus nigra*; *Pinu. virg*., *Pinus virginiana*; *Quer. alba*., *Quercus alba*; *Quer. rubr*., *Quercus rubra*; *Quer. velu*., *Quercus velutina*; *Quer. prin*., *Quercus prinus*; *Frax. amer*., *Fraxinus americana*; *Pinu. stro*., *Pinus strobus*; *Acer. rubr*., *Acer rubrum*; *Acer. sacc*., *Acer saccharum*; *Fagu. gran*., *Fagus grandifolia*; *Tsug. cana*., *Tsuga Canadensis*. (*a*) Growth rate (canopy), (*b*) growth rate (understorey), (*c*) lifespan (canopy), (*d*) lifespan (understorey) and (*e*) per capita reproduction.

There was abundant evidence of demographic differences between the boundaries and the cores of species' ranges. For most rates and most types of boundary, the majority of species showed significant differences in the rate, when compared with the core (*p*<0.05; figure 2). However, the directions and magnitudes of these effects—i.e. the values of *Ω*—varied substantially among the five rates, among species and among boundary types (figure 2).

Nearly all *Ω* values for growth were statistically significant from zero. In part, this reflects the fact that the inventory data contains more information for growth (where each living tree provides a continuous value) than mortality (where each tree either lives or dies). Taking all species together, the *Ω* values for growth rates occupied a narrower range than those for lifespan or per capita recruitment. This implies that, for most species, the boundary–core difference in canopy and understorey growth was substantially less than the difference in canopy or understorey lifespan or per capita recruitment. For example, for canopy growth rate, 50 per cent of the *Ω*(*G*_*L*,*j*_, north) values were in the range −0.22 to −0.08, which corresponds to a 50 per cent range in the proportional difference of canopy growth rate of −19.7 to −8.0 per cent. Analogous ranges for the other growth rates and boundary types were −18.7 to +3.8%, −4.2 to +37.3% and −4.4 to +32.0% for *Ω*(*G*_*D*,*j*_, south), *Ω*(*G*_*L*,*j*_, south) and *Ω*(*G*_*D*,*j*_, south), respectively. Despite being relatively small, the differences in growth were largely consistent among species and boundary type, with most species showing lower growth rates at the northern boundaries and higher growth rates at the southern boundaries (figure 2*a*,*b*, leftmost points). However, three shade-intolerant species showed the opposite pattern, with significantly lower growth rates at the southern boundary. Boundary–core differences in growth were also consistent between the canopy and understorey, with both growth rates showing similar directions and magnitudes of change at a given boundary type (figure 2*a*,*b*, compare black with black and grey with grey).

Lifespan also showed statistically significant differences between the boundary and the core for many species. Perhaps more importantly, the *Ω* values occupied a much larger range than those for growth. The 50 per cent ranges for lifespan were −69.0 to −27.3%, −33.7 to +50.4%, −49.9 to +42.2% and +32.5 to +53.6% for *Ω*(*ρ*_*L*,*j*_, north), *Ω*(*ρ*_*D*,*j*_, north), *Ω*(*ρ*_*L*,*j*_, south) and *Ω*(*ρ*_*D*,*j*_, south), respectively. However, compared to growth, boundary–core differences in lifespan were less consistent among species, boundary type and between the canopy and understorey. An important exception was a consistent trend for decreased canopy lifespan at the northern boundary: 12 of the 19 species showed a statistically significant decrease, whereas only one species showed a significant increase. Averaged across all species, the average reduction in lifespan at the northern boundary was 49.2 per cent.

In contrast to canopy lifespan, understorey lifespan exhibited no trend for a general reduction at the northern boundaries (figure 2; species average *Ω* not significantly different from zero). This reflects the fact that, even within a given species and boundary type, canopy and understorey lifespan often showed contrasting directions and magnitudes of effect. For example, Post oak (*Quercus stellata*) showed a large, significant decrease in canopy lifespan at the northern boundary, but a large, significant increase in understorey lifespan at the same boundary (figure 2). This decoupling of the canopy versus the understorey lifespan contrasts with growth, where the canopy and the understorey rates exhibited very similar patterns for a given species and boundary type (see above). At the southern boundaries, differences in lifespan were highly idiosyncratic among species, with an average *Ω* that was close to zero for both the canopy and understorey; but there were substantial, and often statistically significant, effects within particular species (figure 2).

Compared to the other rates, per capita reproduction $F_{j,R}^{\text{capita}}$ was more difficult to estimate (see the electronic supplementary material). Therefore, the estimates of *Ω* for $F_{j,R}^{\text{capita}}$ should be viewed with some caution, even considering the large confidence intervals (figure 2*e*). Nonetheless, the results suggest some substantial boundary–core differences in $F_{j,R}^{\text{capita}}$, with a 50 per cent range of −58.1 to +201.1% and −67.8 to +7.1% at the northern and southern boundaries, respectively. The results are suggestive of elevated $F_{j,R}^{\text{capita}}$ at the northern boundaries within species of intermediate shade tolerance (figure 2). Overall, however, there was no sign of a consistent difference in reproduction at either boundary type, with the species average *Ω* at both boundaries being not significantly different from zero at *p*<0.05 (figure 2).

### (b) Performance indices

**Figure 3:**
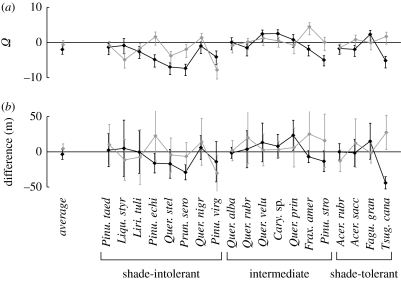
Boundary–core differences in (*a*) *R*_0_ (average number of new recruits produced within the lifetime of an open-grown tree: a measure of rate of spread into an empty landscape) and (*b*) Z^*^ (equilibrium canopy closure height in monoculture: a measure of competitive ability in closed forest). For the definition of *Ω*, error bars, shade tolerance, species order and species abbreviations, see the legend of figure 2. For Z^*^, the difference rather than a log ratio is shown, i.e. Z^*^ in the boundary minus Z^*^ in the core. Black diamonds, northern boundary; grey diamonds, southern boundary.

Boundary–core differences in *R*_0_ for particular species were large, with a 50 per cent range of −99.2 to +6.7% and −80.0 to +240.0% at the northern and southern boundaries, respectively. At the northern boundary, 12 of the 19 species showed a statistically significant reduction in *R*_0_, with only two showing a significant increase. The species average reduction in *R*_0_ at the northern boundary was large in magnitude (−86%), and significantly different from zero. Note that in many respects this result follows that for canopy lifespan at the northern boundary (compare figures 2 and 3). This is to be expected, because canopy lifespan is an important component of *R*_0_ (see above). The species average differences in *R*_0_ at the southern boundary was also negative (average 47% reduction) but not significantly different from zero. However, six species showed a significant difference (five negative and one positive; figure 3*a*). The reduction in *R*_0_ at the northern boundary was more pronounced in early successional species (figure 3*a*). For Z^*^, the confidence intervals on the boundary–core differences were very large, reflecting the fact that the uncertainty in Z^*^ includes the uncertainty in all of the demographic rates on which it depends. Thus, boundary–core differences in Z^*^ were non-significant in most cases (figure 3*b*), and there was no sign of a general pattern according to the boundary type or shade tolerance.

## 4. Discussion

The patterns documented here point to five conclusions with implications for our understanding of, and ability to predict, the geographic distributions of eastern US tree species: (i) for a given species, the northern and southern boundaries of the range typically show substantial differences in demographic rates when compared with the core, and when compared with each other; (ii) these differences occur in growth, mortality and reproduction, and in both the canopy and the understorey; (iii) canopy and understorey growth rates show similar patterns within and among species, being lower at the northern boundaries and greater at the southern boundaries by an average of ±9–17 per cent; (iv) at the northern boundaries canopy lifespan is substantially lower, by an average of 49 per cent; (v) reduced canopy lifespan at the northern boundary reduces *R*_0_ by an average of 86 per cent; and (vi) after accounting for these generalities, there remains a large amount of unexplained interspecific variation in the boundary versus the core demography.

The reduced canopy lifespan, and the reduced canopy and understorey growth, at the northern boundaries documented here, is suggestive of ecological mechanisms contributing to the setting of those boundaries. For example, Loehle (2003) claimed that the northern limits of tree species are set by a trade-off between maximum height growth rate and survival of cold. The fact that the most important of the consistent patterns (in terms of magnitude) was reduced canopy lifespan at the northern boundary supports this claim. More generally, MacArthur (1984) claimed that the northern range boundaries are set by direct environmental limitation, whereas the southern boundaries are set by competition. Of the three general patterns observed at the northern boundaries, two (including the largest in magnitude, i.e. canopy lifespan) referred to the canopy, where the demographic rates are likely to be least affected by competition, being primarily set by the direct interaction between organism and environment. Moreover *R*_0_, a measure of performance in the absence of competition, showed a large, statistically significant reduction at the northern boundary for most species.

By contrast, the results suggest little about the determinants of the southern boundaries, where the analysis revealed no consistent negative effects. The only consistent pattern was a marginally significant trend for increased growth rate (figure 2), as would be expected from the greater annual temperature associated with low latitudes—but this would act to elevate performance in comparison with the core. MacArthur's (1984) hypothesis would appear to imply that, at their southern boundaries, species should exhibit reduced competitive ability. In this analysis, reduced competitive ability would mean lower Z^*^ values, which were not detected at the southern boundaries.

However, in interpreting this and other results, it is helpful to explicitly consider the relationship between density dependence, population dynamics and species ranges. In particular, the intuitive notion that species should exhibit reduced performance near range boundaries does not bear close inspection. To illustrate why, consider the case where the range is at equilibrium, i.e. the range is no longer changing in position or shape and the pattern of abundance within the range is stable. In this case, by definition, each individual within the range must be producing, within its lifetime, one other individual, i.e. lifetime fitness must be equal to 1.0 within all parts of the range. If not, the abundance in some locations, and hence the species range, would still be changing (negative change where lifetime fitness is below 1; positive change where lifetime fitness is above 1).

Such perfect equalization of fitness can only be achieved via density dependence (Chesson 2000). To illustrate how, consider the case where a species begins at very low density everywhere (and for simplicity neglect dispersal processes, source–sink dynamics, fine-scale environmental heterogeneity and interactions with other species). In this case, in locations where lifetime fitness in this situation—i.e. *R*_0_—is less than 1, the species will go locally extinct. Within the remaining locations (i.e. within the range) local abundance will increase, thereby reducing lifetime fitness through density dependence. This increase will continue until lifetime fitness reduces to 1, at which point local abundance will equilibrate and remain stable. Exactly how this density dependence is enacted in trees is not known, except that it is likely to involve effects on all demographic rates. On the other hand, note that if each rate was determined *entirely* by local abundance (i.e. was entirely density-dependent), there could be no dependence of equilibrium abundance on location, i.e. no deterministic structure to the species range. Rather, one or more rates must be subject to density-independent effects, such as the effects of variation in climate and soils. Therefore, in some way, which is yet to be understood, the density-independent and density-dependent effects on each rate of each species studied here (as well as other processes such as source–sink dynamics and species interactions) have combined to determine the abundance of each species at each location observed currently. In addition, species ranges may not be at equilibrium, especially if they are moving northwards in response to recent climate change (Peñuelas *et al*. 2007; Lenoir *et al*. 2008). The demographic patterns documented here reflect the influence of all of these processes.

Therefore, boundary–core differences in demography alone are not sufficient to diagnose the determinants of species ranges. However, they are potentially useful for distinguishing between alternative theories of, and for constraining predictive models of, forest dynamics and biodiversity. For example, at a global scale, the response of biome distributions to climate is a major uncertainty in Earth System Model predictions for climate change (Friedlingstein *et al*. 2006; Sitch *et al*. 2008), implying the need for a new generation of more realistic dynamic global vegetation models (DGVMs). Owing to a lack of data, because they are at an early stage of development, and because of the need to reproduce the current carbon cycle, current DGVMs are built around simple assumptions, some of which are difficult to reconcile with the patterns documented here. For example, DGVMs typically assume that tree lifespan is unresponsive to climate, assume that species within a functional type (e.g. temperate broadleaf) show identical climate dependencies and match the distribution of predicted plant functional types with observations by imposing climate limitations on seedling establishment (e.g. see Foley *et al*. 1996; Sitch *et al*. 2003; Woodward & Lomas 2004; Krinner *et al*. 2005). Similarly, at a regional scale, there is great uncertainty in the potential response of particular species, and hence forest biodiversity and species composition, to climate change. This implies the need for dynamic, process-based alternatives to the static, correlative ‘bioclimate envelope’ models that have been used to estimate these effects to date (Davis *et al*. 1998*a*,*b*). At such time, as these models are developed for the eastern US, an important test of their predictive ability might be the extent to which they can reproduce the generalities and idiosyncrasies of both geographic variation in abundance, as documented previously (Iverson & Prasad 1998; Murphy *et al*. 2006) and geographic variation in demography, as documented here.

## Supplementary Material

The demography of range boundaries versus range cores in eastern US tree species: additional information on methods

Details of FIA data, species selection and statistical methods
