# Supplementary material for: The demography of range boundaries versus range cores in eastern US tree species
Source: Proc Biol Sci. 2009 Feb 25;276(1661):1477–84. doi: 10.1098/rspb.2008.1241 (PMC2677233; doi:10.1098/rspb.2008.1241)
Supplement: The demography of range boundaries versus range cores in eastern US tree species: additional information on methods [file rspb20081241s04.doc]

**The demography of range boundaries vs range cores in eastern US tree species**

Drew W Purves, Microsoft Research Cambridge, UK.

**Supporting material**

Region and study species

The study region is the coterminous United States, east of longitude -105 degrees. The region is approximately 75% forested. The forest communities are predominantly mesic, and composed of a mix of evergreen needleleafs and deciduous broadleafs. The most northerly communities are mainly composed of Boreal species (e.g. Aspens, Spruces); lowland communities at mid-latitudes are dominated by temperate broadleafs (e.g. Oaks, Maples); whereas the communities found in the sandy soils of the southeastern coastal plain are dominated by a mixture of Pines and Oaks. However, the region contains many different forest communities, and within any given community species composition is highly variable. The analysis was restricted to the tree species with >= 10,000 tree records in the FIA data (see below). Several species were not included, either because they were known to have suffered disease outbreaks during the survey interval (*Abies* balsamea), because they are closely managed even in semi-natural forests giving misleading estimates for demographic parameters (*Pinus resinosa*) or because they primarily appear as shrubs rather than trees (several examples). In addition, because the aim of the analysis was to study differences between the boundary and core of the species ranges, any species with the latitudinal center of the range (i.e. midpoint between maximum and minimum latitudes, taken from Burns & Honkala 1990sa, b) outside of the US were excluded. These included some common species, including *Populus tremuloides* and *Pinus banksiana.* This procedure left 19 species remaining (see Fig. 2), which together account for 50% of the tree records in the inventory data. These species showed marked variation in their ranges (e.g. see Fig. 1).

Inventory data

The analysis utilizes the US Forest Service Forest Inventory and Analysis data (FIA), a network of small permanent forest inventory plots that covers most of the coterminous US. These plots are surveyed on a regular basis. For more information see Smith (2002), McRoberts *et al.* (2005). The major advantages of the FIA are (1) plots are located randomly within forested regions; (2) the same plots are revisited, meaning that observations of the appearance, growth, and death, of individual trees are available; (3) the large size of the database (see below).

The FIA data available for this study were from plots surveyed once in the 1980s, and again in the 1990s, with the exact years differing from state to state. For the time period in question, a number of variables were recorded for each plot, including time since stand-levelling disturbance, and plantation vs. natural forest. Within each plot, trees were sampled from a cluster of circular plots, with radius varying according to state and tree size (see Hanson *et al.* 1992, Purves *et al.* 2004). For each tree sampled, a number of observations were recorded, including species, status (live, dead from harvesting, dead from natural causes) and diameter at breast height (dbh). The analysis was restricted to include only those plots that were recorded as forested at both times; that were recorded as non-plantation origin at both times; that had no recorded tree harvesting between the two survey dates; and that underwent no stand-level disturbance between the two survey dates.

A key part of the analysis was to distinguish the demographic rates rates of canopy vs subcanopy trees (see below). For trees that were alive at the time of the second survey, the FIA data provided a recorded crown class for each tree at the time of the second survey, which could be used to infer canopy status (i.e. in or out of the canopy) at the time of the second survey. However, crown class was not available for the time of the first survey, nor for trees that died between the two survey dates. Because of this, for parameter estimation I used a probabilistic approach, assigning to each tree *i* a value $P_{i}$, defined as the *probability* that tree *i* was in the canopy at the time of the first survey. To calculate $P_{i}$, I used FIA crown-class data reported for the second survey, to calculate $P_{i}$ for each species *j*, conditional on the *prediction* of canopy status (in or out of the canopy) given by the Ideal Tree Distribution (ITD) model described in Purves *et al*. (2007). Applied to the second survey. [Note that Purves *et al.* 2004 employed two different parameter estimation schemes for the crown shape parameters, from which I employed the ‘single axis fit’ parameters here]. That is, we used the ITD model to generate a value of $Z^{*}$ (height of canopy closure) for each inventory plot *q* at the time of the second survey, and then assigned a predicted canopy status $U_{i}^{(pred)}$ for the time of the second survey (1= canopy, 0 = understory) to each tree *i* in *q* ($U_{i}^{(pred)}$= 1 if tree *i* is taller than $Z^{*}$, and 0 otherwise: see Purves *et al.* 2007). I then compared the observed canopy status $U_{i}^{(obs)}$ of the trees in *q* at the time of the second survey with the predictions $U_{i}^{(pred)}$ to give the conditional probabilities:

$P_{i,t}$ = $\gamma_{1,j}$ if $U_{i,t}^{(pred)}=1$

$\gamma_{0,j}$ if $U_{i,t}^{(pred)}=0$ (S1)

where *t* refers to the time of the survey (1 = first survey; 2 = second survey), and

$$\gamma_{1,j}=\frac{S[U_{i,2}^{(obs)}=1 \text{and} U_{i,2}^{(pred)}=1]}{S[U_{i,2}^{(pred)}=1]}$$

$\gamma_{0,j}=\frac{S[U_{i,2}^{(obs)}=1 \text{and} U_{i,2}^{(pred)}=0]}{S[U_{i,2}^{(pred)}=0]}$ (S2)

where *S*[] denotes the number of trees *i* that match the criteria contained in []. Typical values of $\gamma_{1,j}$ and $\gamma_{0,j}$ were 0.90 and 0.20 respectively, showing that the ITD model gave quite accurate predictions of canopy status for individual trees.

The same approach to estimating canopy- vs understory parameters was used with FIA data from the Lake States in (Purves *et al.* 2008), providing model parameters that led to accurate predictions of 100-year forest dynamics.

Definition of species rangeS

The range of each species *j* was broken into regions *R*, where *R* is a set of 0.5 x 0.5 degree grid-cells *k*. The definition of the regions *R* was specific to each species *j*. The purpose of the regions was to divide the species ranges into a ‘core’ region, and several ‘boundary’ regions (north, south, or the entire boundary). Importantly, the analysis did not make any *a priori* assumptions about the nature or shape of the species’ ranges. For example, it did not assume circular ranges, or assume that the greatest abundance occurs at the center of the range. Rather, as described below, it was designed in a way that allowed naturally for the irregular, sometimes disjoint, ranges observed in the data (see Fig 1).

Defining the regions *R* for species *j* began by calculating $B^{(j,k)}$, which is the average basal area (m^2^ ha^-1^) of species *j* within forest stands in 0.50 x 0.50 degree grid-cell *k*:

$B^{(j,k)}=\left(\frac{1}{C_{plots}^{(k)}}\right)\sum_{ q\in S(k)} B^{(j,q,1)}$ (S3)

where the set $S(k)$ contains all inventory plots *q* within grid-cell *k* ; $C_{plots}^{(k)}$ is the number of plots in this set; and $B^{(j,q,1)}$ is the basal area (m^2^ ha^-1^) of species *j* in plot *q* at the time of the first survey:

$B^{(j,q,1)}=\sum_{i\in T(j,q,1)} w^{(i)}B^{(i)}$ (S4)

where $B^{(i)}$ is the basal area (m^2^) of tree *i* at the time of the first survey; the set $T(j,q,1)$ contains all trees within plot *q* with species identity *j* that were recorded as alive at the time of the first survey; and where $w^{(i)}$ (ha^-1^) is an expansion factor for tree *i*, which places all trees on an equal per-area basis (in this case, per hectare). The $w^{(i)}$ values were needed here because the sampling radius used in the inventory plots varied according to plot design (which varied from state to state), and according to tree size (variable radius plot sampling). For more information on expansion factors see Purves *et al.* (2004).

To define the regions *R* for species *j*, step 1 was to discard any grid-cell *k* with $B^{(j,k)}$ = 0. Step 2 was a spatial smoothing of the remaining$B^{(j,k)}$ values, to help compensate for sampling uncertainty in the$B^{(j,k)}$for any given grid-cell *k*. This step consisted of applying the following transformation to each grid-cell *k*:

(S5)

where the set $Z(k)$ contains the eight neighbouring grid-cells of *k*. After this transformation, $B^{(j,k)}$ became a weighted average of the data from the plots within grid-cell *k*, and the plots in the grid-cells neighbouring *k*. Where *k* had a full set of eight neighbours, the weighting was equal between$B^{(j,k)}$, and all neighbouring cells combined (see the 1/8 value in eq S5).

Finally, step 3 was to rank the grid-cells containing species *j* according to the smoothed values of $B^{(j,k)}$. Each grid cell *k* was then assigned to one of three *abundance bands*, according to whether it appeared in the first third of the ranked list (abundance band 0), the second third (abundance band 1), or the final third (abundance band 2). A similar approach was used to assign each grid-cell to a *latitude band*. I.e., the grid-cells containing *j* were ranked by latitude, with each cell being assigned a latitude band depending on whether it appeared in the first third of this list (band 0, most northerly third of cells), the second third (band 2) or the final third (band 3, most southerly third of cells). Thus, each grid-cell containing species *j* was assigned one of three abundance bands for *j*, and one of three latitude bands.

Then, each grid-cell *k* was assigned to one or more regions *R*, depending on the combination of abundance band and latitude band assigned to *k.* The *core* consisted of all grid-cells in abundance band 0, regardless of latitude band; the northern boundary consisted of all grid-cells that lay both within abundance band 2, and latitude band 0; the southern boundary consisted of all grid-cells that lay both within abundance band 2 and latitude band 2; and the entire boundary consisted of any grid-cell in abundance band 2, regardless of latitude band.

There are two principal benefits of this approach. First, it uses observed spatial variation in abundance, rather than distance from the center (or edge) of a species range, to define the relevant boundary. This is important, because the ranges of many species are highly irregular, such that Euclidean distance from the range center would be expected to have little relevance to the species biology. Second, because species become rarer toward range boundaries, the sample size available for a given species in a given grid-cell *k* decreases toward the range boundaries. This means that reliably estimating parameters for particular locations, for particular species, is impossible except for locations within or near the core of the range. But, dividing the range into a small number of discrete regions, as explained above, and grouping all available data within each band or region, provides large sample sizes for estimating rates within each region.

ESTIMATING RATES

For each species *j* and region *R*, the following rates were estimated from the inventory data: diameter growth rate for canopy trees ($G_{L,j,R}$: units cm yr^-1^); growth rate for understory trees ($G_{D,j,R}$: units cm yr^-1^); lifespan of canopy trees ($\rho_{L,j,R}$: units yrs); lifespan of understory trees ($\rho_{D,j,R}$: units yrs); and per-capita reproduction (*F:* units yr^-1^ m^-2^ ha^1^). Lifespan does not imply that all individuals live for a set period. Rather, lifespan refers to the reciprocal of the annual mortality rate. I.e., the analysis assumes that canopy trees are subject to a constant, size- and age-independent probability of dying each year. The reciprocal of this rate is the average lifespan. Lifespan was used here rather than the annual mortality rate for the sake of interpretation: using lifespan means that an increase in any of the estimated parameters implies increased fitness.

We used the Metropolis-Hastings Markov chain Monte Carlo (MCMC) algorithm, within a Bayesian context, to generate estimates for the posterior probability distributions for each parameter, given the inventory data for species *j* within region *R*. For a given parameter, the posterior density is proportional to the product of the likelihood (calculated from data) and the prior (Gelman *et al.* 2004). However, we used uniform priors on a finite range for each parameter. With uniform priors, the posterior distribution for each parameter depends only on the likelihood. This, together with the large sample sizes in this analysis, imply that in this case, the parameter estimates obtained from Bayesian analysis should be very similar to those that would have been obtained using Maximum Likelihood methods.

For each species *j* and region *R*, three separate Bayesian analyses were carried out: one of growth, one for lifespan, and one for reproduction. In each case, all that was necessary was to define the log-likelihood function , which returns the logarithm of the likelihood of the data $\mathbf{X}$, given a particular vector of parameters $\mathbf{\theta}$. The likelihood functions for each of the three analyses are given below. Given the likelihood function, Metropolis-Hastings MCMC sampling was then used to estimate the posterior distribution of $\mathbf{\theta}$, given $\mathbf{X}$. From these posteriors, the posterior mean, posterior standard deviation, and 68% credible intervals, for each parameter of interest were extracted from the samples returned by the MCMC algorithm. The statistical methodology followed here is very similar to that used in Purves *et al* (2008), the supporting information for which includes a slightly expanded discussion of some technical details listed here. For more information about Bayesian analysis and MCMC sampling, see Gelman *et al.* (2004).

GROWTH

For each species *j* and region *R,* an MCMC algorithm was used to estimate posterior distributions of four growth parameters: the growth rate (cm yr^-1^) of canopy trees $G_{L,j,R}$, and understory trees $G_{D,j,R}$, and the standard deviations (cm yr^-1^) $\sigma_{L,j,R}$ and $\sigma_{D,j,R}$, which describe the magnitude of the unexplained variation in the growth rates of canopy and understory trees respectively. For estimation using MCMC, it was found to be more stable numerically to define $G_{D,j,R}$ as a logit function of $G_{L,j,R}$ as follows: $G_{D,j,R}=(1/[1+\exp(-\lambda_{j,R})])G_{L,j,R}$ , where $\lambda_{j,R}$ is a parameter that sets $G_{D,j,R}$ as a fraction of $G_{L,j,R}$. The parameters estimated by the MCMC algorithm were then $G_{L,j,R}$, $\lambda_{j,R}$, $\sigma_{L,j,R}$ and $\sigma_{D,j,R}$. Note that this approach carries the assumption that the understory growth rate is lower than the canopy growth rate.

The growth rates of trees were assumed to be independent of each other, such that the likelihood was described by

=

$\sum_{i\in\mathbf{X}_{growth,j,R}} \ln\left\{P_{i}N(\Delta D_{i}|G_{L,j,R},\sigma_{L,j,R})+(1-P_{i})N(\Delta D_{i}|G_{D,j,R},\sigma_{D,j,R})\right\}$ (S6.1)

$G_{D,j,R}=(1/[1+\exp(-\lambda_{j,R})])G_{L,j,R}$ (S6.2)

where $P_{i}$ is the probability that tree *i* was in the canopy at the time of the first survey (see above), and $N(\Delta D_{i}|G_{L,j,R},\sigma_{L,j,R})$ is the normal probability density for the observed growth rate of tree *i,* $\Delta D_{i}$, given the mean $G_{L,j,R}$ and standard deviation $\sigma_{L,j,R}$. Equation S6.1 represents a sum over all trees *i* within the set $\mathbf{X}_{growth,j,R}$, which contains all trees of species *j* in region *R* that were measured at both survey times, and recorded as alive at both survey times. The observed growth rate $\Delta D_{i}$ was calculated as $[dbh_{2,i}-dbh_{1,i}]/\Delta T_{i}$ where $dbh_{1,i}$ and $dbh_{2,i}$ are the diameter at breast height (cm) of tree *i* at the time of the first survey and second survey respectively, and $\Delta T_{i}$ is the survey interval (years) for tree *i*.

The MCMC algorithm returned 2500 vectors {$G_{L,j,R}$, $\lambda_{j,R}$, $\sigma_{L,j,R}$,$\sigma_{D,j,R}$} drawn from the posterior distribution of these parameters. These vectors were used to generate a set of 2500 samples of $G_{D,j,R}$ using error propagation, as follows. The values of $G_{L,j,R}$ and $\lambda_{j,R}$ in each vector was used to calculate a value of $G_{D,j,R}$, giving 2500 samples for $G_{D,j,R}$. For both parameters $G_{L,j,R}$ and $G_{D,j,R}$, the mean and standard deviation of the 2500 samples was calculated (these were used in a further analysis step: see BOUNDARY:CORE RATIOS below). The top and bottom 16% of the samples of each parameter were then discarded, the range of the remaining samples forming 68% credible intervals. Here and elsewhere, 68% intervals were used because they are analogous to standard deviations (i.e. in a normal distribution, the mean ± one standard deviation contains 68% of the distribution). Also, the true value $P'$ of any parameter *P* being below the 68% interval on *P* is 0.16, and true value $P'$ of any parameter *P* being above the 68% interval on *P* is 0.16. Thus, two parameters with 68% intervals that just overlap are statistically different at *p* ≈ 0.16^2^ = 0.026, providing that the intervals on the parameters are similar. Thus, any two parameters with 68% intervals that do not overlap can be viewed as having means that are significantly different at *p* < 0.05.

LIFESPAN

For each species *j* and region *R,* an MCMC algorithm was used to estimate posterior distributions of two lifespan parameters: the expected lifespan (years) of canopy trees$\rho_{L,j,R}$, and understory trees $\rho_{D,j,R}$, where the lifespans $\rho_{L,j,R}$ and $\rho_{D,j,R}$ are the reciprocal of the annual mortality rates (probability of dying in a given year) $\mu_{L,j,R}$ and $\mu_{D,j,R}$, i.e., $\rho_{L,j,R}=1/\mu_{L,j,R}$ and $\rho_{D,j,R}=1/\mu_{D,j,R}$. In addition, for estimation using MCMC, it was found to be more stable numerically to define $\rho_{D,j,R}$ as a logit function of $\rho_{L,j,R}$ as follows: $\rho_{D,j,R}=(1/[1+\exp(-\varphi_{j,R})])\rho_{L,j,R}$ , where $\varphi_{j,R}$ is a parameter that sets $\rho_{D,j,R}$ as a fraction of $\rho_{L,j,R}$. Note that this approach carries the assumption that the understory lifespan is lower than the canopy lifespan.

The survival of different trees was assumed to be independent, such that the likelihood was described by

=

$\begin{matrix} \sum_{i\in\mathbf{X}_{mort,j,R}} \ln\left\{M_{i}\left[(1-P_{i})(1-(1-\mu_{D,j,R})^{\Delta T_{i}})+P_{i}(1-(1-\mu_{L,j,R})^{\Delta T_{i}})\right]\right. \\ \left.+(1-M_{i})\left[(1-P_{i})(1-\mu_{D,j,R})^{\Delta T_{i}}+P_{i}(1-\mu_{L,j,R})^{\Delta T_{i}}\right]\right\} \end{matrix}$ (S7.1)

$$\mu_{L,j,R}=1/\rho_{L,j,R}$$

$\mu_{D,j,R}=1/\rho_{D,j,R}$ (S7.2)

$$\rho_{D,j,R}=(1/[1+\exp(-\varphi_{j,R})])\rho_{L,j,R}$$

where $P_{i}$ is the probability that tree *i* was in the canopy at the time of the first survey (see above), $\Delta T_{i}$ is the survey interval (years) for tree *i*, and $M_{i}$ is the observed mortality of tree *i* (1 = died, 0 = survived). Equation. S7.1 represents a sum over all trees *i* within the set $\mathbf{X}_{mort,j,R}$, which contains all trees of species *j* in region *R* that were measured at both survey times, and recorded as alive at the time of the first survey.

The MCMC algorithm returned 2500 vectors {$G_{L,j,R}$, $\varphi_{j,R}$} drawn from the posterior distribution. To calculate a posterior distribution for $\rho_{D,j,R}$, propagation of error was employed, as follows. The values of $\rho_{L,j,R}$ and $\varphi_{j,R}$ in each of these vectors was used to calculate a value of $\rho_{D,j,R}$ for each vector, giving 2500 samples for $\rho_{D,j,R}$. For both parameters $\rho_{L,j,R}$ and $\rho_{D,j,R}$, the mean and standard deviation of the 2500 samples was calculated (these were used in a further analysis step: see BOUNDARY:CORE RATIOS below). The top and bottom 16% of the samples for each parameter were then discarded, the range of the remaining samples forming 68% credible intervals.

REPRODUCTION

The per-capita reproductive rate for species *j,* region *R*, $F_{j,R}^{capita}$ was defined as the number of new trees produced per year in region *R*, per unit basal area (m^2^ ha^-1^) of species *j* in the forests within region *R*. Therefore, the parameter $F_{j,R}^{capita}$ was estimated in two steps. First, the absolute recruitment rate (trees per hectare per year) $F_{j,R}^{absolute}$ was estimated (along with a shape parameter $k_{j,R}$: see below), using the observed rate of appearance of new individuals of species *j* in the inventory plots in region *R*. Recruitment into different plots was assumed to be independent, and the observed rate of appearance of new individuals in each plot represents the sum over multiple years. Therefore, I assumed a negative binomial distribution for the number of new recruits observed in the data, such that the likelihood was defined by

=

$\sum_{q\in\mathbf{X}_{recr,j,R}} \ln\left\{ negbinom[\right.V_{j,q},E\{V_{j,q}\},k_{j,R}] \}$ (S8.1)

$E\{V_{j,q}\}=F_{j,R}^{absolute}.\Delta T_{q}.A_{q}^{recr}$ (S8.2)

where $V_{j,q}$ is the observed number of new recruits of species *j* in inventory plot *q*; $E\{V_{j,q}\}$ is the expectation for $V_{j,q}$ given the mean arrival rate per year $F_{j,R}^{absolute}$, interval $\Delta T_{q}$ and the area (ha) of plot *q* surveyed for new recruits, $A_{q}^{recr}$; and $negbinom[V_{j,q},F_{j,R}^{absolute},k_{j,R}]$ is a negative binomial probability density for $V_{j,q}$, given the mean $E\{V_{j,q}\}$ and shape parameter $k_{j,R}$. Note that this procedure required the estimation of two parameters: $F_{j,R}^{absolute}$ and the shape parameter $k_{j,R}$. Equation. S8.1 represents a sum over all plots *q* within the set $\mathbf{X}_{recr,j,R}$, which contains all plots in region *R* that were measured at both survey times. The observed recruitment for $V_{j,q}$ in plot *q* is the number of trees in plot *q* that were recorded as present at the time of the second survey, but not recorded at the time of the first survey, not counting false recruitment events (‘on growth’, the apparent sudden appearance of large trees due to variable radius plot sampling; and false events due to the addition of recruitment survey plots at the time of the second survey).

Given the likelihood defined in Eq. S8, the MCMC algorithm returned 2500 samples from the posterior probability distributions for $F_{j,R}^{absolute}$ and $k_{j,R}$. From these samples were calculated the mean and standard deviation of $F_{j,R}^{absolute}$. This mean and standard deviation were then used to calculate $F_{j,R}^{capita}$ using error propagation, as follows. First, 1000 samples of $F_{j,R}^{capita}$ were generated. Each sample was generated using $F_{j,R}^{capita,sample}=n(F_{j,R}^{absolute})/n(B_{j,R})$ where $n(F_{j,R}^{absolute})$ is a random sample from a normal distribution with mean and standard deviation taken from the posterior on $F_{j,R}^{absolute}$ (see above), and $n(B_{j,R})$ is a random sample from a normal distribution with mean and standard error of the basal area (m^2^ ha^-1^) of species *j* in region *R.* [Standard error was used here because it corresponds to the uncertainty in the mean basal area, in the same way that the standard deviation from the posterior of the parameter $F_{j,R}^{absolute}$ represents the uncertainty in the mean recruitment rate $F_{j,R}^{absolute}$ ]. From this set of 1000 samples were calculated the mean and standard deviation on $F_{j,R}^{capita}$ (these were used in a further analysis step: see BOUNDARY:CORE RATIOS below). The top and bottom 16% of the samples for each parameter were then discarded, the range of the remaining samples forming a 68% credible interval on $F_{j,R}^{capita}$.

Please note that the estimates for $F_{j,R}^{capita}$ are made difficult by the fact that the FIA includes only trees with dbh >= 2.54 cm. Thus, by the time a cohort of new recruits appears in the data, it has already gone through a relatively long period of growth and mortality. Therefore the apparent $F_{j,R}^{absolute}$ is lower than its true value, because the loss of saplings is not accounted for. Thus, the estimates of $F_{j,R}^{absolute}$ are subject to an unknown negative bias. However, note also that, in calculating the $\Omega$ values for $F_{j,R}^{capita}$ (see below), the bias would be partially corrected because it appears in both the numerator and denominator. Thus, the $\Omega$ values for reproduction are subject to an unknown source of error, depending on the *difference* *in the bias* between the boundary and core. This *difference* in bias is likely to be less than the bias in the estimates of $F_{j,R}^{capita}$ for a given region.

BOUNDARY: CORE RATIOS

The procedures detailed above returned, for each species *j* and region *R*, the posterior mean and standard deviation for each of five key demographic parameters: the growth rates $G_{L,j,R}$, $G_{D,j,R}$, the lifespans $\rho_{L,j,R}$ and $\rho_{D,j,R}$, and per-capita (i.e. per basal area) reproductive rate $F_{j,R}^{capita}$. Error propagation was then used to measure the proportional difference in each of these parameters between the core, and three types of boundary: the northern boundary, the southern boundary, and the entire boundary. The procedure is illustrated here for $G_{L,j,R}$, but was identical for $G_{D,j,R}$, $\rho_{L,j,R}$,$\rho_{D,j,R}$, and $F_{j,R}^{capita}$.

The values$\Omega(G_{L,j},all)$, $\Omega(G_{L,j},north)$, $\Omega(G_{L,j},south)$were defined as the ratio of $G_{L,j,R}$ in the boundary, to that in the core, for the entire boundary (‘*all*’), and the northern and southern boundaries (‘*north*’, ‘*south’*), with the ratio expressed as a natural logarithm. To estimate the $\Omega$ values, whilst accurately representing the uncertainty in the demographic parameters within any one region, the error propagation generated 10,000 samples of each $\Omega$ value:

$\Omega^{sample}(G_{L,j},all)=\ln\{n(G_{L,j,entire\_boundary})/n(G_{L,j,core})\}$ (S9.1)

$\Omega^{sample}(G_{L,j},north)=\ln\{n(G_{L,j,northern\_boundary})/n(G_{L,j,core})\}$ (S9.2)

$\Omega^{sample}(G_{L,j},north)=\ln\{n(G_{L,j,southern\_boundary})/n(G_{L,j,core})\}$ (S9.3)

where $n(G_{L,j,core})$ is a random sample from a normal distribution with mean and standard deviation equal to that taken from the posterior for $G_{L,j,core}$, and similarly for $n(G_{L,j,entire\_boundary})$, $n(G_{L,j,northern\_boundary})$ and $n(G_{L,j,southern\_boundary})$. From each of these sets of 10,000 samples, the mean was calculated. The largest and smallest 2.5% of values of values were then discarded, the range on the remainder providing a 95% credible interval on the value of $\Omega$.

Note that, because of the definition of the $\Omega$ values and the error propagation method used, the interpretation of the results of the analysis are as follows: (1) a mean $\Omega$, for species *j*, of above zero implies that the most likely difference between the boundary and core is that the rate is higher, for species *j*, in the boundary; (2) a mean $\Omega$, for species *j*, of below zero implies that the most likely difference between the boundary core is that the rate is lower, for species *j*, in the boundary; (3) a 95% credible interval on $\Omega$ that does not include the zero represents a statistically significant difference (*p* < 0.05) between the boundary and the core, in whichever direction.

**Performance indices: $R_{0}$ and** $Z^{*}$

The performance indices $R_{0}$ and $Z^{*}$ are overall measures of performance, calculated using several of the demographic rates discussed above (see main text). For species *j* in region *R* (not to be confused with the *R* in $R_{0}$), the value of $R_{0}$ could be calculated as:

$R_{0,j,R}$ $=(1/10000).F_{j,R}^{capita}.\int_{\tau=0}^{\infty} \exp(-[1/\rho_{L,j,R}]\tau).\pi.([G_{L,j,R}\tau/2])^{2} d\tau$ (S10.1)

$=(1/10000).F_{j,R}^{capita}.(\pi/2).G_{L,j,R}^{2}.\rho_{L,j,R}^{3}$ (S10.2)

Eq. S10.1 is an integral over the lifetime of the tree, with the first term inside the integral being the probability that the tree is still alive at time $\tau$ , and the second term giving the tree’s basal area (in cm^2^) at time $\tau$. The correction term 1/1000 is needed because $F_{j,R}^{capita}$ refers to basal area in m^2^. Note that this calculation assumes that the growth, and mortality, rates for the tree remain constant during the entire lifetime of the tree.

The index $Z^{*}$ (hereafter subscripts *j* and *R* are omitted) is defined as the canopy closure height in an equilibrium monoculture (Purves *et al.* 2008). This index comes from the PPA model (Strigul *et al.* 2008, Purves *et al.* 2008). This model assumes that there exists a critical height $Z^{*}$, above which any foliage experiences direct sunlight, and below which all light has already been filtered by the canopy. The procedure for calculating the equilibrium value of $Z^{*}$, denoted $\hat{Z}^{*}$ (the hat was left off in the main text for convenience) depends on the particular assumptions made within the PPA model, which is a flexible formulation allowing for different rules governing growth, mortality, and competition for canopy space. To calculate $\hat{Z}^{*}$ in this case, I utilized a very simple version of the PPA model, used in Adams *et al.* (2007) and Purves *et al.* (2008). The key assumptions of this special case are that all trees have potential crowns consisting of a flat disc expressed at the top of the tree; that canopy trees exhibit a constant diameter growth rate and constant mortality rate; and that understory trees exhibit a constant diameter growth rate and constant mortality rate. For more details see Purves *et al.* (2008).

The first step to calculating $\hat{Z}^{*}$, for this special case of the PPA, is to recognize that, with a fixed dbh-height allometry, $\hat{Z}^{*}$ corresponds to a critical diameter $\hat{D}^{*}$:

$$Z^{*}=\alpha[\hat{D}^{*}]^{\beta}$$

where the parameters $\alpha$ and $\beta$ set the height allometry. The second step is to recognize that, where $\hat{D}^{*}$ is not varying through time, each new cohort of trees begins in the understory, and passes through the diameter $\hat{D}^{*}$ after a time *T* years, where the value of *T* is set by the growth rate in the understory *T* = $\hat{D}^{*}/G_{D,j,R}$. During this time, the trees in the cohort die at a rate $1/\rho_{D}$, such that the proportion of the trees that make it to the canopy, denoted $P^{canop}$ here, is $\exp(-(T/\rho_{D}))=\exp(-[\hat{D}^{*}/(G_{D}\rho_{D})])$. Because, in this special case of the PPA, it is assumed that only canopy trees can produce seeds, only this fraction of each cohort goes on to even begin to produce seeds.

The second step is to calculate the lifetime reproductive value for those trees that do reach the canopy, denoted here $R^{canop}$:

(S11)

where eq. S11 represents an integral over all possible times $\tau$ after the tree entered the canopy. The value of the term in the integral is a product of the basal area at time $\tau$(set by the growth rate in the canopy), the probability that the tree is still alive at time $\tau$(set by the mortality rate in the canopy), and the number of new recruits produced, per year, per unit basal area (set by the parameter $F^{capita}$). The correction term 1/1000 is needed because $F_{j,R}^{capita}$ refers to basal area in m^2^. A more convenient approximation to $R^{canop}$ is provided by disregarding the diameter of the tree when it first enters the canopy (which is equal to $\hat{D}^{*}$: the initial diameter accounts for only a small fraction of the total fecundity under most reasonable parameter values):

$R^{canop}\approx\int_{\tau=0}^{\infty} F^{capita}(1/10000)\pi\left(\frac{G_{L}\tau}{2}\right)^{2}\exp(-[\tau/\rho_{L}]) d\tau$ (S12.1)

$\approx(1/10000).F^{capita}.(\pi/2).G_{L}^{2}.\rho_{L}^{3}$ (S12.2)

Note that this implies that $R^{canop}\approx R_{0}$ (see above).

The third step is to solve for $\hat{D}^{*}$, which is done by setting the lifetime reproductive value of the cohort to 1 (because this condition is necessary for the population to be at equilibrium):

$P^{canop}R^{canop}\approx\exp(-[\hat{D}^{*}/(G_{D}\rho_{D})]).(1/10000).F^{capita}.(\pi/2).G_{L}^{2}.\rho_{L}^{3}=1$ (S13.1)

$\hat{D}^{*}\approx[G_{D}.\rho_{D}][\ln((\pi/2)(1/10000)F^{capita}G_{L}^{2}\rho_{L}^{3})]$ (S13.2)

Finally, the value of $\hat{D}^{*}$ (a stem diameter) is converted to a tree height $\hat{Z}^{*}$, using the height allometry:

$Z^{*}\approx\alpha(\hat{D}^{*})^{\beta}$ (S14.1)

$Z^{*}\approx\alpha[G_{D}.\rho_{D}]^{\beta}[\ln((\pi/2)(1/10000)F^{capita}G_{L}^{2}\rho_{L}^{3})]^{\beta}$ (S14.2)

Where the parameters $\alpha$ and $\beta$ determine the height allometry. In this case, values of $\alpha$ and $\beta$ determined for the most common eastern US tree species, Red Maple *Acer rubrum*, were used ($\alpha$=4.49, $\beta$=0.455) for all species. The choice of $\alpha$ and $\beta$ would be expected to have little impact on results in this case, because $\alpha$ cancels when calculating proportional differences in $\hat{Z}^{*}$, and because $\beta$ varies little among species.

**References for supporting material**

Gelman,A., Carlin, J.B., Stern, H.S., Rubin, D.B. (2004). *Bayesian Data Analysis. Second Edition.* Chapman & Hall, Boca Raton, Florida.

Hansen, M.H., T. Frieswyck *et al.*, *USDA Forest Service General Technical Reports* **NC-151** (1992).

McRoberts, R.E., Bechtold, W.A., Patterson, P.L., Scott, C.T., and G.A. Reams. 2005. The enhanced forest inventory and analysis program of the USDA Forest Service: Historical perspective and announcement of statistical documentation. *Journal of Forestry* **103**: 304 – 308.

Smith, W.B. 2002. Forest inventory and analysis: a national inventory and monitoring program. Environmental Pollution **116:** S233 – S242, Suppl 1.

**Purves DW**, Caspersen JP, Moorcroft PR, Hurtt GC, Pacala SW (2004). Human-induced changes in U.S. biogenic VOC emissions: evidence from long-term forest inventory data. *Global Change Biology*, **10**, 1737 - 1755.

**Purves, DW**, Lichstein, JW & Pacala, SW. (2007). Crown plasticity and competition for canopy space: a spatially implicit model parameterized for 250 North American tree species*. PLoS-ONE* **2(9)**: e870. doi:10.1371/journal.pone.0000870

**Purves, DW**, Lichstein, JW, Strigul, N, Pacala, SW (2008 *in press*). Predicting and understanding forest dynamics using a simple tractable model. *Proceedings of the National Academy of Science USA.*
